# Imagerie du volvulus du grêle sur mésentère commun incomplet chez un adulte: rapport de cas

**DOI:** 10.11604/pamj.2020.37.287.17538

**Published:** 2020-11-30

**Authors:** Amina Alaoui, Badreddine Alami, Youssef Alaoui Lamrani, Mariam Boubou, Mustapha Maaroufi

**Affiliations:** 1 Service de Radiologie, CHU Hassan II, Fes, Maroc

**Keywords:** Volvulus, mésentère commun, occlusion intestinale, intestin grêle, rapport de cas, Small bowel volvulus, common mesentery, intestinal occlusion, case report

## Abstract

Le diagnostic du volvulus du grêle peut se faire en urgence devant un tableau d´occlusion intestinale aiguë, voire un état de choc ou devant des douleurs abdominales répétées associées souvent à des troubles du transit. C´est une complication redoutable du mésentère commun incomplet, qui est définie comme une anomalie de rotation du tube digestif très rare à l´âge adulte. Sa symptomatologie clinique est non spécifique, d´où l´intérêt de connaitre ses caractéristiques radiologiques surtout scannographiques, permettant ainsi une prise en charge thérapeutique précoce. Nous rapportons l´observation d´un patient de 65 ans admis pour volvulus total du grêle sur mésentère commun incomplet diagnostiqué par tomodensitométrie (TDM) abdominale et confirmé par l´exploration chirurgicale, et chez qui l´évolution était favorable.

## Abstract (en)

Emergency diagnosis of small bowel volvulus can be established in patients with acute intestinal obstruction, or even shock, or repeated abdominal pain often associated with motility disorders. This is a life-threatening complication of common incomplete mesentery, which is defined as an intestinal malrotation occurring very rarely in adults. Clinical symptoms are non-specific, hence the importance of knowing the radiological features, in particular scannographic features of this rare entity, thus enabling early therapeutic management. We here report the case of a 65-year-old patient admitted with total small bowel volvulus complicating common incomplete mesentery, diagnosed based on abdominal computed tomography (CT) scan and confirmed at surgery, who had favorable outcome.

## Introduction

Le mésentère commun résulte d'une anomalie de rotation du tube digestif á 180° [1]. Il s´agit d´une malformation congénitale du mésentère exceptionnelle à l´âge adulte (0,2% à 0,5%), de symptomatologie variable et source de multiples erreurs et retard diagnostiques et thérapeutiques au point que la majorité des cas sont diagnostiqués en post-mortem [2]. La complication la plus redoutable reste le volvulus total du grêle, qui se traduit cliniquement par syndrome occlusif. L´imagerie joue un rôle incontournable dans le diagnostic. Dans la pratique courante on commence par un abdomen sans préparation (ASP) qui va mettre en évidence des niveaux hydro-aériques de type grêlique en rapport avec le syndrome occlusif, toutefois il ne permet pas d´orienter le diagnostic étiologique, ce qui amène à réaliser une TDM qui confirme l´occlusion et permet d´assoir le diagnostic de volvulus en montrant le signe du « tourbillon » qui semble être pathognomonique pour la majorité des auteurs [3]. Le volvulus total du grêle sur mésentère commun incomplet est une urgence chirurgicale, la procédure de LADD reste le traitement de référence aussi bien chez l'adulte que chez l'enfant [1].

## Patient et observation

**Figure 1:**
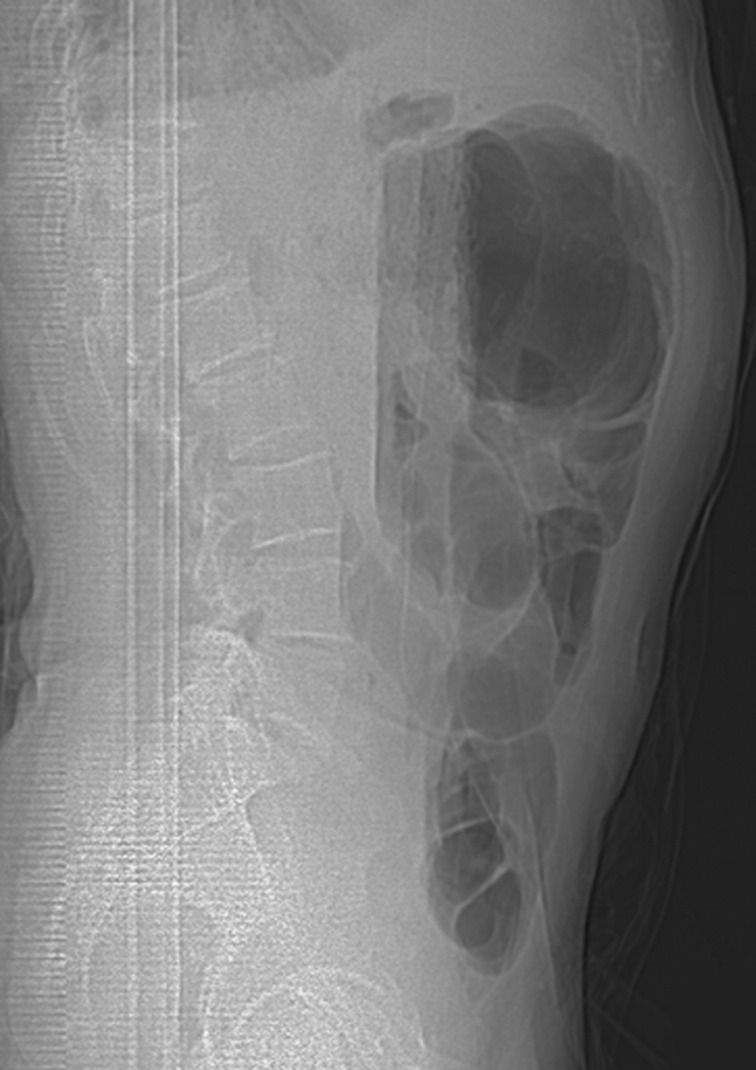
ASP profil en position couchée: montre des niveaux hydro-aériques centraux plus larges que hauts de type grêlique

**Figure 2:**
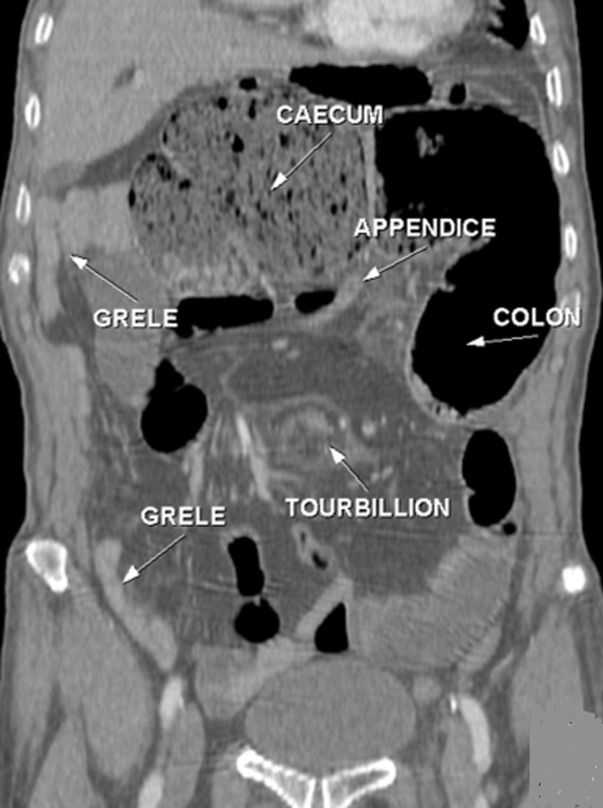
TDM abdominale en reconstruction coronale: montre la présence d´une distension grêlique en amont d´une image en tourbillon en rapport avec un volvulus avec positionnement anormal du grêle à droite alors que le cæcum et l´appendice sont en sous hépatique

**Figure 3:**
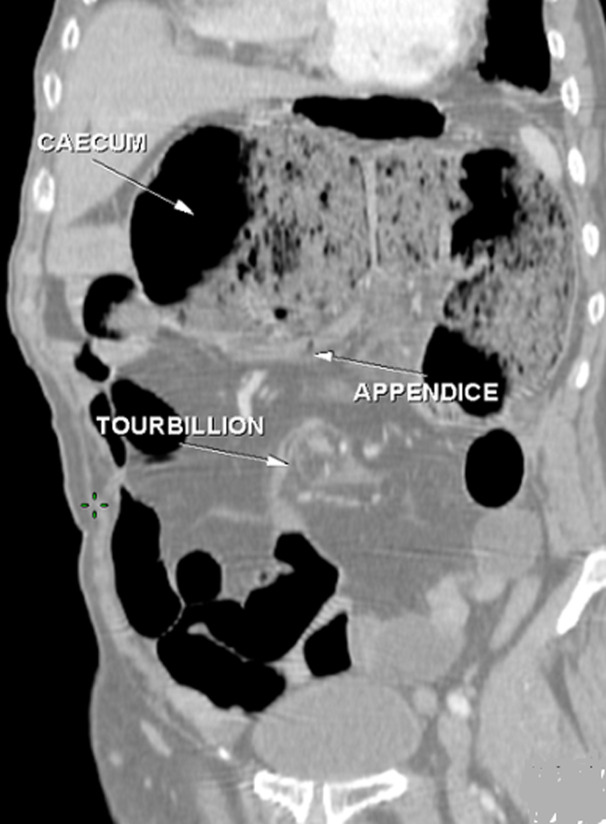
TDM abdominale en reconstruction axiale: montre la présence d´une distension grêlique en amont d´une image en tourbillon en rapport avec un volvulus avec positionnement anormal du grêle à droite alors que le cæcum et l´appendice sont en sous hépatique

**Figure 4:**
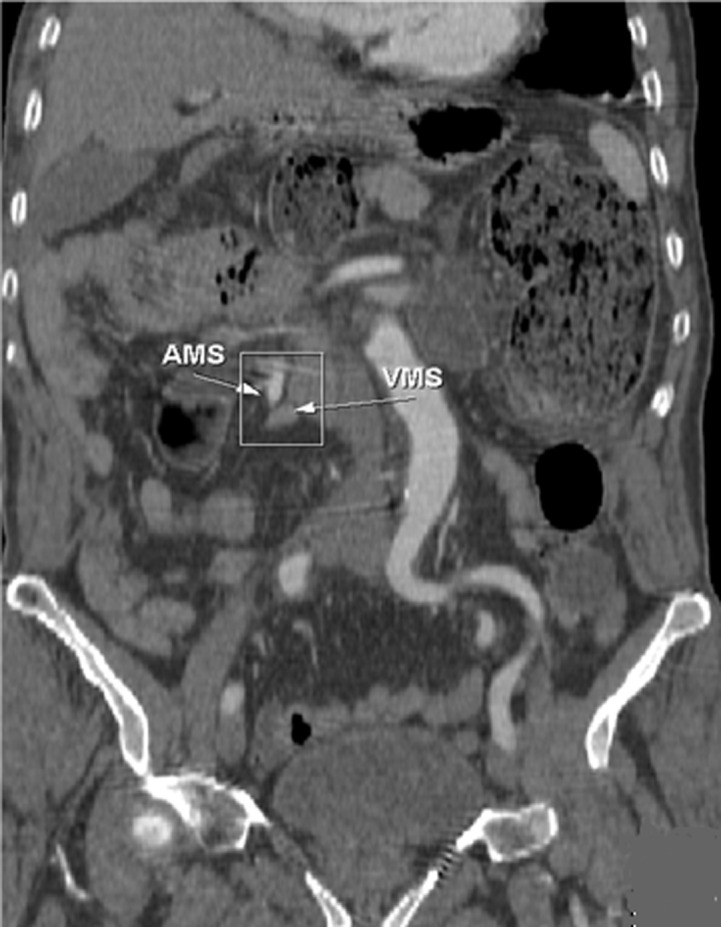
TDM abdominale en reconstruction coronale: montre une verticalisation de l´artère et la veine mésentériques supérieures

**Figure 5:**
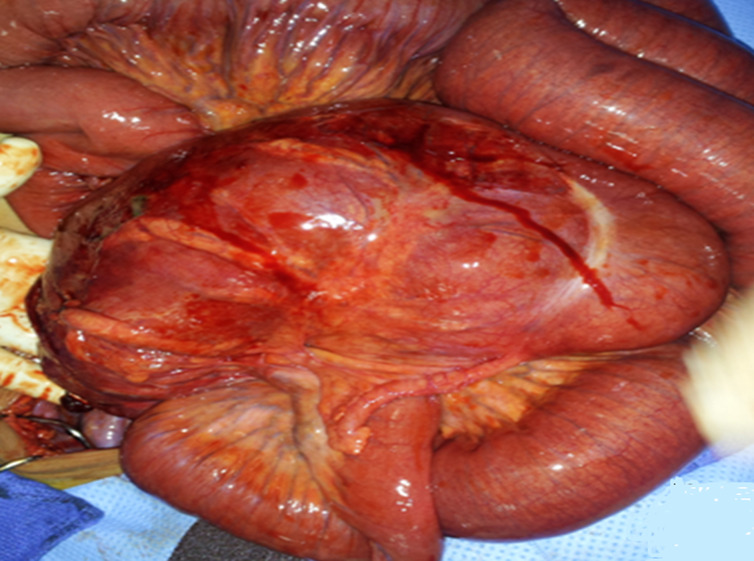
image per-opératoires du volvulus du grêle sur mésentère commun incomplet

Il s´agit d´un patient de 65 ans, admis aux urgences pour un syndrome occlusif fait d´arrêt des matières et des gaz remontant à une semaine. L´examen clinique a trouvé un patient eupnéique, apyrétique avec un abdomen distendu, tympanique et sans anomalie à la palpation des orifices herniaires ou au toucher rectal. Le bilan biologique (NFS, CRP, Ionogramme) était normal. L´ASP (Figure 1) a montré des niveaux hydro-aériques centraux plus larges que hauts de type grêlique. Une TDM abdomino-pelvienne réalisée en urgence, a objectivé la présence d´une distension grêlique en amont d´une image en tourbillon en rapport avec un volvulus (Figure 2, Figure 3), associée à verticalisation de l´artère et de la veine mésentériques supérieures (Figure 4) et un positionnement anormal du grêle à droite alors que le cæcum et l´appendice sont en sous hépatique (Figure 2, Figure 3). D´après l´analyse de l´ensemble des images tomodensitométriques le diagnostic du volvulus du grêle sur mésentère commun incomplet a été évoqué. Ce qu´a été confirmé à l´exploration chirurgicale (Figure 5) qui a consisté à une dévolvulation manuelle du grêle avec découverte d´une anse souffrante sans nécrose pariétale, ensuite une cure de l'anomalie de rotation a été réalisé selon la procédure de LADD. Les suites post opératoires ont été simples.

## Discussion

Le mésentère commun résulte d'une anomalie de rotation du tube digestif [1]. Le fait que cette pathologie soit exceptionnelle à l´âge adulte et que sa symptomatologie soit assez variée est source de multiples erreurs et de retard diagnostique et thérapeutique au point que la majorité des cas sont diagnostiquées en post-mortem [2]. La prévalence de ces malformations congénitales à l´âge adulte est estimée à l'ordre de 0,2% à 0,5% [4,5]. Et la complication la plus redoutable est le volvulus total du grêle. L´arrêt de la rotation intestinale à 180° aboutit à une ascension du cæcum vers le haut et la droite; préduodénal. La dernière anse grêle est donc proche de l´angle duodéno-jéjunal; et par conséquent, cette disposition est la plus fréquemment rencontrée lors des volvulus, où la rotation est quasiment toujours horaire (inverse au sens de rotation normal). On note souvent la présence d´une bride entre le cæcum et la paroi abdominale supéro-latérale droite. Cette bride péritonéale est nommée communément « Bride de Ladd »; bien connue des chirurgiens. Elle croise le deuxième duodénum et peut être responsable d´une occlusion intestinale aiguë haute chez un patient adulte auparavant asymptomatique. Parfois, il peut même exister un accolement congénital entre le méso de ces deux anses intestinales (« fusion mésentérique de Pellerin ») [1,6].

Dans cette position à 180°, la racine du mésentère est extrêmement courte et l'ensemble de l'intestin grêle se trouve « pédiculé » sur son axe vasculaire mésentérique supérieur. Cette position, dite en « mésentère commun incomplet », est à haut risque de volvulus total du grêle du fait de la brièveté de la racine du mésentère et de son absence d'accolement [6]. Sur le plan clinique, le mésentère commun incomplet non compliquée est souvent asymptomatique et le diagnostic peut se faire dans des circonstances très variées: de manière fortuite au cours d'un examen radiologique ou rarement au décours d´une chirurgie laparoscopique [7,8]. Comme il peut se manifester par un tableau de douleurs abdominales répétées plus ou moins associées à des troubles du transit [9]. Le volvulus total du grêle constitue la complication la plus redoutable de cette anomalie de rotation et le diagnostic se fait en urgence, devant un tableau d'occlusion intestinale aiguë, voir un état de choc pouvant conduire au décès.

En pratique courante, devant tel tableau clinique chez un adulte, il est essentiel de penser à évoquer précocement le diagnostic du volvulus du grêle sur mésentère commun incomplet, afin d'être en mesure de le confirmer, idéalement en préopératoire, par un examen tomodensitométrique [10]. Ce dernier constitue le gold standard pour le diagnostic positif, topographique et de gravité. Techniquement c´est une acquisition abdomino-pelvienne avec injection de produit de contraste. Le signe du « tourbillon » semble être pathognomonique pour la majorité des auteurs [11], décrit par Fisher [12] en 1981 sous le nom de whirl-like pattern, il correspond à la vrille du mésentère visible en position médiane, en avant de l'aorte et au niveau de l'artère mésentérique supérieure, autour de laquelle viennent « s'enrouler » la veine mésentérique supérieure et le jéjunum proximal. L´injection du produit de contraste permet de visualiser la verticalisation, ou l'inversion, des vaisseaux mésentériques supérieurs, avec une veine se plaçant au-dessus ou à gauche de l'artère [13], bien que ce signe ne soit pas constant. L'épaisseur de cette masse de tourbillon serait proportionnelle au degré de rotation du volvulus, mais il est plus précis d'évaluer le degré de rotation en calculant le nombre de spires réalisés par les vaisseaux mésentériques [14].

L'abdomen sans préparation (ASP) est l´examen de 1^ère^ intention devant un tableau d´occlusion intestinale aiguë, qui peut mettre en évidence des niveaux hydro-aériques de type grêlique comme il peut ne montre aucun signe spécifique du volvulus sur mésentère commun incomplet, cependant il est rarement normal et généralement interpréter comme « inhabituel » ou discordant. L´échographie doppler chez l´adulte est souvent gênée par les gaz et n'est pas toujours contributive au diagnostic, cependant sa sensibilité serait de 86,5%, sa spécificité de 74,7%, sa valeur prédictive positive de 42,1% et sa valeur prédictive négative de 96,3% [15]. La connaissance de l'anatomie du mésentère commun incomplet est indispensable pour en faire le diagnostic en peropératoire et comprendre les principes de sa cure chirurgicale. Le volvulus total du grêle sur mésentère commun incomplet est une urgence chirurgicale, la procédure de LADD reste le traitement de référence aussi bien chez l'adulte que chez l'enfant [1]. Cette procédure consiste d´abord en une laparotomie médiane suivie d'une réduction du volvulus par détorsion dans un sens antihoraire, ensuite, on procède à une section des brides responsables du raccourcissement de la racine mésentérique puis une fixation de l'intestin au mésentère commun pour éviter toute récidive. La procédure comporte aussi une appendicectomie de principe [3]. L´évolution est généralement favorable, à condition d´une prise en charge à temps, avant l´installation d´une souffrance digestive.

## Conclusion

La tomodensitométrie abdomino-pelvienne avec injection du produit de contraste est le gold standard pour poser le diagnostic positif, topographique et de gravité du volvulus du grêle sur mésentère commun incomplet chez l´adulte. Toutefois la réalisation d'examen radiologique ne doit pas retarder la prise en charge thérapeutique. Le pronostic du volvulus total du grêle est celui du syndrome occlusif, et dépend fortement du délai de prise en charge et du terrain.
